# Evaluation of Parents’ Knowledge and Attitudes Towards Pediatric Dental Practice during the COVID-19 Pandemic

**DOI:** 10.3290/j.ohpd.b1248969

**Published:** 2021-04-22

**Authors:** Koray Surme, Hayri Akman, Leyla Cime Akbaydogan, Mehmet Akin

**Affiliations:** a Assistant Professor, Department of Pediatric Dentistry, Faculty of Dentistry, Alanya Alaaddin Keykubat University, Antalya, Turkey. Designed the study, analysed the data, wrote the manuscript.; b Assistant Professor, Department of Pediatric Dentistry, Faculty of Dentistry, Alanya Alaaddin Keykubat University, Antalya, Turkey. Designed the study, collected the data, wrote the manuscript.; c Assistant Professor, Department of Orthodontics, Faculty of Dentistry, Alanya Alaaddin Keykubat University, Antalya, Turkey. Designed the study, collected the data, analysed the data.; d Associate Professor, Department of Orthodontics, Faculty of Dentistry, Alanya Alaaddin Keykubat University, Antalya, Turkey. Collected the data.

**Keywords:** COVID-19, pediatric dentistry, cross-infection control, dental treatment

## Abstract

**Purpose:**

The coronavirus disease 2019 (COVID-19) pandemic is a major public health crisis worldwide and it also has generated new challenges for dentistry. The aim of this study was to evaluate the knowledge and attitudes of the parents of pediatric patients about dental treatment during the COVID-19 pandemic through a questionnaire.

**Materials and Methods:**

A structured questionnaire consisting of 15 multiple-choice questions and demographic information about the knowledge and attitudes of parents regarding dental treatment during the COVID-19 outbreak was used for the study. The participants were parents of pediatric patients (aged 8–14 years) who visited for a routine orthodontic examination at the department of orthodontics.

**Results:**

A total of 250 participants responded to the questionnaire. The findings indicate that more than 95% of parents had information about the transmission paths of the virus, took COVID-19 seriously, and told their children about this disease. 34% of the parents thought that dental clinics were more dangerous than other social areas, and 39.2% thought their children could be infected by medical instruments during dental treatment. A statistically significant difference was observed between educational levels in the answers given about the transmission paths of the virus, the danger of dental clinics in terms of the virus, the permitted dental treatment procedures, and the personal protective equipment of the dentist (p < 0.05).

**Conclusion:**

Although most parents have information about COVID-19, there are differences in the knowledge and attitudes of parents during the pandemic period according to their educational level.

In December 2019, severe acute respiratory syndrome coronavirus (SARS-CoV) created an emergency in Wuhan, China.^6^ Soon the disease called coronavirus disease 2019 (COVID-19) began to affect the whole world, and The World Health Organization (WHO) declared a pandemic on March 11, 2020.^18^ According to data published by the World Health Organization (WHO) up to December 24, 2020, COVID-19 has affected more than 200 countries and territories, with a total of more than 76 million confirmed cases and more than 1.7 million deaths worldwide. In Turkey, the virus has infected more than 1.2 million people and a total of 18,602 people have died from COVID-19.^20^

The most common symptoms of COVID-19 are weakness, muscle pain, dry cough, fever, shortness of breath, and breathing difficulties. Sometimes sore throat and diarrhea can be observed. While the symptoms are mild in most patients, 15% of the patients require hospitalisation and 5% of them require intensive care.^4^ Although all people are susceptible to COVID-19, the symptoms of the disease in children are generally milder and have a better prognosis. Children who are asymptomatic are usually diagnosed due to an anomaly on a chest CT.^16^ Although symptomatic COVID-19 patients are the main source of transmission, asymptomatic patients with an incubation period of approximately 1 to 14 days can also transmit the virus. Moreover, it has been reported that virus transmission is possible even during the recovery period of patients.^12^ We must be attentive to children who can carry the disease without showing symptoms, in order to prevent the spread of the virus.

Due to the nature of dental treatment, the dentist has to work close to the patient and aerosols are present in many routine dental procedures. During treatment procedures, the dentist may be exposed to the patient’s blood and other body fluids. In addition, it has been reported that COVID-19 can survive in aerosol for up to 3 h; on different surfaces such as plastic and stainless steel, it can survive up to 72 h, albeit in small amounts.^19^ Therefore, dentists are in the high-risk group in terms of COVID-19 transmission. Providing treatment to patients is important, but the main goal should be to prevent the transmission of infection to patients and dental healthcare professionals.^11^ To prevent transmission of the disease to other patients and healthcare personnel, every pediatric patient should be handled as potentially COVID-19 positive and maximum personal protective equipment (PPE) should be used. Generally, FFP2 or FFP3 mask, face shield, cap, gloves and waterproof disposable gowns are used as PPE in dentistry.^8^

During the COVID-19 pandemic, patients have been exposed to a tremendous amount of information from various publications and social media, including that the virus can be transmitted when patients attend their appointments at dental clinics or hospitals. It is important to find out whether the patients have any been misinformed during this period. The aim of this study was to evaluate the knowledge and attitudes of the parents of patients about dental treatment during the COVID-19 pandemic through a questionnaire.

## Materials and Methods

The questionnaire study was conducted on parents who referred to the Faculty of Dentistry, Alanya Alaaddin Keykubat University in August and September 2020. The participants were parents of pediatric patients (aged 8–14 years) who visited for a routine orthodontic examination at the department of orthodontics. Pediatric patients with systemic diseases were not included in the study because their parents might have worries about dental treatment.

The study was approved by the local ethics committee (20/22-17). A consent form was given to the participants explaining the purpose of the research. All parents signed written consent to participate in the study. The questionnaires were filled in by the parents in the waiting room before the orthodontic appointment of the participant’s child. To ensure privacy and confidentiality, individual results were not made public in any way.

We developed a questionnaire to obtain information about the knowledge and attitudes of parents of pediatric patients regarding infection control procedures in dental care during the COVID-19 pandemic. Since there are limited studies about COVID-19 and parents, a questionnaire was designed and modified from questionnaires about other infectious diseases used in previous similar studies.^3,15,17^ The questions were validated and pilot-tested to assess clarity. The questions were evaluated by four experts (two pediatric dentists, one orthodontist, and one biostatistician) prior to the study to assess the content adequacy of the questionnaire and how clear the statements of the items were. Based on the comments made by these experts, the final version of the questionnaire was designed as 15 questions and demographic information. The questionnaire initially designed was first given to 20 individual parents to determine whether the questions were clear and understandable. After the completion of each questionnaire, it was asked if there were any questions or difficulties encountered while completing it and parents commented on the questionnaire.

The final questionnaire included 15 questions, including information about sex and age of children and parents, parents’ educational status, knowledge level about COVID-19 pandemic (ways to access information, pay attention, whether s/he could explain COVID-19 to the child, paths of transmission), parents’ attitudes about infection-control procedures in dental treatment during the COVID-19 pandemic period (Table 1). 276 parents were asked to participate in the questionnaire. Questionnaires with missing answers to at least one question were excluded from the study.

**Table 1:** Questionnaire evaluating the knowledge and attitudes of parents regarding dental procedures during the COVID-19 period

Questions	Answers
Q1. Do you have information about Covid-19 and its transmission paths?	a. Yes
	b. No
Q2. If your answer to the previous question is yes, how did you get this information? (You can mark one or more.)	a. I read or watched the public spotlights*
	b. I watched programs about COVID-19 on TV
	c. I got information from social media platforms such as WhatsApp, Facebook and Instagram
	d. I watched a presentation, conference or webinar
	e. I got information from my friends or family
Q3. Do you take the COVID-19 seriously?	a. Yes, I take seriously
	b. No, I think it’s exaggerated
Q4. Have you told your child about the COVID-19?	a. Yes
	b. No
Q5. Do you think dental clinics are more dangerous than other social areas?	a. Yes, it is more dangerous
	b. It is similarly dangerous
	c. No, it is less dangerous
Q6. Do you think dental treatment can infect your child with Covid-19?	a. Yes
	b. I think it is as dangerous as social life
	c. No
Q7. If your child had dental treatment, in what ways do you think he/she could be infected during this time?	a. Droplets and blood
	b. Medical instruments
	c. From the dentist or assistant
Q8. If your child had a toothache during the Covid-19 pandemic, would you go to the dental clinic?	a. Yes
	b. No
Q9. If your answer to the previous question is yes, which of the following treatments you agree to have?	a. Only examination
	b. Only examination and tooth extraction
	c. All operations including all necessary restorative treatments
Q10. What kind of gown should the dentist wear?	a. Civil dress
	b. Scrubs
	c. White coat over a civil dress
	d. Disposable gown
	e. Isolation gown
	f. It does not matter
Q11. Which of the following protective equipment should the dentist use during treatment in the Covid-19 pandemic? (You can mark one or more.)	a. Mask
	b. Glove
	c. Goggles
	d. Face shield
	e. Hair cover
Q12. What do you think about the dentist’s use of personal protective equipment during your child’s treatment?	a. The use of personal protective equipment bothers me as it may frighteh my child
	b. It makes me feel safe as it can prevent COVID-19 transmission
	c. Whether the doctor uses protective equipment or not is not important to me
Q13. What do you think personal protective equipment is used for? (You can mark one or more.	a. To avoid cross infection from dentist to patient
	b. To avoid cross infection from patient to dentist
	c. To avoid cross infections from other patients
	d. For psychological and personal reasons
Q14. Would you prefer your child to wear protective glasses during treatment?	a. Yes
	b. No
Q15. Would you prefer your dentist to cover medical equipment (suction tip, air water syringe, dental unit, dental curing light, etc) with plastic wrap?	a. Yes
	b. No

*Information bulletin from the Ministry of Health.

### Statistical Analysis

IBM SPSS 21.0 software (IBM; Armonk, NY, USA) was used for data analysis. Descriptive statistical methods were employed, and the chi-squared test was used to assess statistically significant differences between respondents according to their education level. The statistical significance level was set at p < 0.05.

## Results

Of the 276 parents who were asked to participate in the questionnaire, 21 refused to participate and 5 answered incompletely. 250 parents answered the questionnaire in full. Demographic information about the age, gender, and educational levels of the parents and their children is shown in Table 2. Male parents were 46.8% (n = 117) of the total number, while female parents were 53.2% (n = 133). 41.2% (n = 103) of the children were male and 58.8% (n = 147) were female. 94 (37.6%) of the parents were primary school graduates, 82 (37.6%) were high school graduates, and 74 (37.6%) were university or college graduates. The chronological ages of the parents and children were 42.62 ± 6.24 years, 12.36 ± 2.03 years, respectively.

**Table 2:** Demographic information of parents and children

		Parents’ educational level			
		Primary school	High school	College or university	Total
Parent’s gender	Male n (%)	42 (44.7%)	34 (41.5%)	41 (55.4%)	117 (46.8%)
	Female n (%)	52 (55.3%)	48 (58.5%)	33 (44.6%)	133 (53.2%)
Child’s gender	Male n (%)	38 (40.4%)	36 (43.9%)	29 (39.2%)	103 (41.2%)
	Female n (%)	56 (59.6%)	46 (56.1%)	45 (60.8%)	147 (58.8%)
Parent’s age	Mean age and SD (y)	43.35 ± 6.82	41.31 ± 5.73	43.12 ± 5.71	42.62 ± 6.24
Child’s age	Mean age and SD (y)	12.55 ± 1.99	12.28 ± 1.83	12.2 ± 2.28	12.36 ± 2.03

n: sample size; SD: standard deviation; y: year.

96.4% of the parents had information about the transmission paths of the virus and they obtained this information mostly from ‘public spotlights’ (information bulletins from the Ministry of Health) and TV. 99.6% of parents took COVID-19 seriously, and 97.6% told their children about this disease. About half of the parents thought that dental clinics were as dangerous as other social areas, and that dental treatment posed similar risks to social life in terms of infecting their child with COVID-19. 39.2% of the parents thought that their children could be infected by medical instruments during dental treatment. While 76.8% of the parents said that they would go to the dentist if their children had a toothache, about half of them said they would only allow examination and tooth extraction. In addition, 63.2% of the parents preferred the dentist to use disposable gowns. They chose all the options at similar rates on the question of which PPE equipment the dentist should use. Almost all of the parents thought that using PPE during dental treatment of their children would make them feel safe. While the majority of the parents stated that PPE use should be used to prevent cross-infection between patients and dentists, only 1.6% of them thought that PPE should be used for psychological and personal reasons. About half the parents preferred that their children wear protective glasses during dental treatment, but 73.2% of the parents stated that they preferred the medical devices to be covered with plastic wrap (Table 3).

**Table 3:** Frequencies and percentages of answers, comparison of parents’ answers by their educational levels

Questions	Answers	Educational level of parents				Chi-squared and p-values
		Primary school	High school	College or university	Total	
Q1	a	85 (90.4%)	82 (100%)	74 (100%)	241 (96.4%)	χ^2^ test=15.494 p=0.000***
	b	9 (9.6%)	0 (0%)	0 (0%)	9 (3.6%)	
	Total	94 (37.6%)	82 (32.8%)	74 (29.6%)	250 (100%)	
Q2	a	54 (31%)	68 (29.3%)	62 (27.4%)	184 (29.1%)	χ^2^ test=11.513 p=0.174
	b	62 (35.6%)	70 (30.2%)	60 (26.5%)	192 (30.4%)	
	c	26 (14.9%)	46 (19.8%)	44 (19.5%)	116 (18.4%)	
	d	12 (6.9%)	16 (6.9%)	30 (13.3%)	58 (9.2%)	
	e	20 (11.5%)	32 (13.8%)	30 (13.3%)	82 (13.0%)	
	Total	174 (27.5%)	232 (36.7%)	226 (35.8%)	632 (100%)	
Q3	a	93 (98.9%)	82 (100%)	74 (100%)	249 (99.6%)	χ^2^ test=1.703 p=0.427
	b	1 (1.1%)	0 (0%)	0 (0%)	1 (0.4%)	
	Total	94 (37.6%)	82 (32.8%)	74 (29.6%)	250 (100%)	
Q4	a	92 (97.9%)	78 (95.1%)	74 (100%)	244 (97.6%)	χ^2^ test=3.999 p=0.135
	b	2 (2.1%)	4 (4.9%)	0 (0%)	6 (2.4%)	
	Total	94 (37.6%)	82 (32.8%)	74 (29.6%)	250 (100%)	
Q5	a	23 (24.5%)	25 (30.5%)	37 (50.0%)	85 (34.0%)	χ^2^ test=15.668 p=0.003**
	b	52 (55.3%)	48 (58.5%)	29 (39.2%)	129 (51.6%)	
	c	19 (20.2%)	9 (11.0%)	8 (10.8%)	36 (14.4%)	
	Total	94 (37.6%)	82 (32.8%)	74 (29.6%)	250 (100%)	
Q6	a	21 (22.3%)	18 (22.0%)	24 (32.4%)	63 (25.2%)	χ^2^ test=7.710 p=0.103
	b	46 (48.9%)	38 (46.3%)	40 (54.1%)	124 (49.6%)	
	c	27 (28.7%)	26 (31.7%)	10 (13.5%)	63 (25.2%)	
	Total	94 (37.6%)	82 (32.8%)	74 (29.6%)	250 (100%)	
Q7	a	24 (25.5%)	28 (34.1%)	30 (40.5%)	82 (32.8%)	χ^2^ test=4.389 p=0.356
	b	41 (43.6%)	32 (39.0%)	25 (33.8%)	98 (39.2%)	
	c	29 (30.9%)	22 (26.8%)	19 (25.7%)	70 (28.0%)	
	Total	94 (37.6%)	82 (32.8%)	74 (29.6%)	250 (100%)	
Q8	a	76 (80.9%)	58 (70.7%)	58 (78.4%)	192 (76.8%)	χ^2^ test=2.054 p=0.358
	b	18 (19.1%)	24 (29.3%)	16 (21.6%)	58 (23.2%)	
	Total	94 (37.6%)	82 (32.8%)	74 (29.6%)	250 (100%)	
Q9	a	38 (50.0%)	18 (31.0%)	14 (24.1%)	70 (36.5%)	χ^2^ test=15.390 p=0.004**
	b	6 (7.9%)	2 (3.4%)	9 (15.5%)	17 (8.9%)	
	c	32 (42.1%)	38 (65.5%)	35 (60.3%)	105 (54.7%)	
	Total	76 (39.6%)	58 (30.2%)	58 (30.2%)	192 (100%)	
Q10	a	0 (0%)	0 (0%)	0 (0%)	0 (0%)	χ^2^ test=30.922 p=0.000***
	b	4 (4.3%)	10 (12.2%)	6 (8.1%)	20 (8.0%)	
	c	2 (2.1%)	0 (0%)	2 (2.7%)	4 (1.6%)	
	d	66 (70.2%)	50 (61.0%)	42 (56.8%)	158 (63.2%)	
	e	10 (10.6%)	6 (7.3%)	22 (29.7%)	38 (15.2%)	
	f	12 (12.8%)	16 (19.5%)	2 (2.7%)	30 (12%)	
	Total	94 (37.6%)	82 (32.8%)	74 (29.6%)	250 (100%)	
Q11	a	88 (28.0%)	78 (25.0%)	74 (24.3%)	240 (25.8%)	χ^2^ test=4.044 p=0.853
	b	80 (25.5%)	72 (23.1%)	74 (24.3%)	226 (24.3%)	
	c	45 (14.3%)	54 (17.3%)	57 (18.8%)	156 (16.8%)	
	d	63 (20.1%)	62 (19.9%)	59 (19.4%)	184 (19.8%)	
	e	38 (12.1%)	46 (14.7%)	40 (13.2%)	124 (13.3%)	
	Total	314 (33.8%)	312 (33.5%)	304 (32.7%)	930 (100%)	
Q12	a	2 (2.1%)	0 (0.0%)	0 (0.0%)	2 (0.8%)	χ^2^ test=7.661 p=0.105
	b	91 (96.8%)	82 (100.0%)	71 (95.9%)	244 (97.6%)	
	c	1 (1.1%)	0 (0.0%)	3 (4.1%)	4 (1.6%)	
	Total	94 (37.6%)	82 (32.8%)	74 (29.6%)	250 (100%)	
Q13	a	80 (33.3%)	78 (35.5%)	60 (36.6%)	218 (34.9%)	χ^2^ test=19.269 p=0.004**
	b	88 (36.7%)	73 (33.2%)	64 (39.0%)	225 (36.1%)	
	c	62 (25.8%)	69 (31.4%)	40 (24.4%)	171 (27.4%)	
	d	10 (4.2%)	0 (0.0%)	0 (0.0%)	10 (1.6%)	
	Total	240 (38.5%)	220 (35.3%)	164 (26.3%)	624 (100%)	
Q14	a	44 (46.8%)	40 (48.8%)	41 (55.4%)	125 (50.0%)	χ^2^ test=1.297 p=0.523
	b	50 (53.2%)	42 (51.2%)	33 (44.6%)	125 (50.0%)	
	Total	94 (37.6%)	82 (32.8%)	74 (29.6%)	250 (100%)	
Q15	a	68 (72.3%)	59 (72.0%)	56 (75.7%)	183 (73.2%)	χ^2^ test=0.332 p=0.847
	b	26 (27.7%)	23 (28.0%)	18 (24.3%)	67 (26.8%)	
	Total	94 (37.6%)	82 (32.8%)	74 (29.6%)	250 (100%)	

Q: question. *p < 0.05, **p < 0.01, ***p < 0.001.

A statistically significant difference was observed between the answers given to the questions 1, 5, 9, 10 and 13 according to the educational levels of the parents (p < 0.05). However, there was no statistically significant difference between the answers given to the other questions (p < 0.05) (Table 3).

## Discussion

COVID-19 is a viral infectious disease and can be transmitted rapidly from person to person. Therefore, the control and prevention of infection transmission has received more focus in dentistry recently. In this study, a questionnaire was used to evaluate the knowledge and attitudes of the parents towards dental treatments during the COVID-19 pandemic. Although there are many studies on the attitude of patients regarding cross-infection transmission in dentistry, since COVID-19 is a very new disease, there is a very limited number of studies on the attitude of patients or parents to COVID-19 transmission.^7,15,17^ To our knowledge, this is the first study in Turkey to evaluate the knowledge and attitudes of parents during the COVID-19 pandemic.

Individuals should be aware of public health policies concerning basic infection control protocols to be implemented. In addition, educating the public about the COVID-19 and raising public awareness play a vital role in the control and prevention of the pandemic.^13^ Our study shows that parents have a very high rate of knowledge about the transmission paths of COVID-19 and take the disease seriously. In a previous study, it was reported that patients had a good amount of knowledge about the paths of transmission of COVID-19.^2^ However, 9.6% of parents with a primary school education stated that they did not have information about the transmission paths of COVID-19. A statistically significant difference was found between the educational levels of parents according to their level of knowledge about the paths of transmission (p < 0.05).

In a previous study conducted in Jordan, parents were asked how they got information about COVID-19, showing that patients received information mostly from news channels, social media, and the Ministry of Health website.^1^ Similar to these findings, in our study, it was found that parents mostly received information from TV programs, public spotlights prepared by the Ministry of Health, and social media. While it is possible to access more accurate information from TV programs and public spotlights, sometimes misinformation is obtained through social media. Almost all of the parents who participated in this questionnaire explained COVID-19 to their children; this rate needs to be high to individually protect children from the disease.

It has long been reported that viruses and bacteria can be transmitted to both patients and healthcare professionals in dental practice.^5^ In a previous study, a total of 91.9% of the parents answered that their children could be easily infected with the virus while receiving dental treatment.^17^ In contrast, in this study, 25.2% of parents stated that their children could be infected with COVID-19 during dental treatment. This difference may be due to the difference of a few months between the two studies. To reduce the spread of COVID-19, it has been recommended to provide only urgent dental treatments and online consultation.^22^ During the COVID-19 pandemic period, the majority of parents answered ‘yes’ to the question ‘would you visit the dentist if your child had a toothache?’, but a noteworthy rate of 23.2% declared that even if their children had a toothache, they would not visit dental clinics. 54.7% of the parents who stated that they would go to the dentist during this period in case of toothache also agreed to have all dental procedures, including root canal treatment and fillings, while 8.9% of the parents stated that they would only consent to tooth extraction; 36.5% of them would only consent to an examination. These results show that a significant portion of the parents who bring their children to dental clinics may be hesitant about having all dental procedures. Parents whose formal education ended with graduation from primary school mostly marked the option that they would only have an examination, while highschool and university graduates marked the option that they would allow all restorative procedures. Their answers were statistically significantly different (p < 0.05) according to their educational level.

Although droplets were reported to be the most important transmission path of COVID-19, in this study, parents thought their children could be infected at a similar rate from medical instruments or healthcare professionals themselves.^14^ Since aerosols and droplets are formed in dental settings, the risk of cross-infection between dentists and patients may be high if necessary precautions are not taken; therefore, effective infection control protocols should be implemented in dental practices and hospitals especially during the COVID-19 pandemic.^10^ It is thought that many children only show mild, inconspicuous symptoms of COVID-19 and thus increase the chances of becoming unidentified carriers.^9^ To reduce the transmission risk of infection, every child should be evaluated as potentially infected in regions where the disease incidence is high. It is recommended that dental practitioners use of personal protective equipment, such as FFP2 or FFP3 filter face masks, gloves, gowns, goggles, hair covers, and face shields for protection from aerosols and droplets.^10^ In this study, parents mostly preferred that dentists use disposable gowns, and stated that other PPE should also be used. It has been reported that when dental professionals wear a colorful uniform, it may lead to a decrease in anxiety levels in children seeking dental treatment.^21^ In contrast, in this study, 97.6% of the parents stated that the dentist’s use of PPE would make them feel safe. This difference may be due to the parents’ anxiety about COVID-19 transmission.

Parents generally stated that PPE should be used to prevent cross-infection between patients and dentists. 4.2% of parents with a primary school education stated that PPE should be used for personal and psychological reasons; there is a statistically significant difference between educational levels in terms of the answers given to this question. This difference may be due to the preference of parents who are primary school graduates for dentists to use PPE for psychological and personal reasons. 50% of the parents stated that their children should wear protective glasses during treatment, and 73.2% of the parents preferred that the medical instruments be covered with plastic wrap. Covering dental instruments with plastic wrap can make parents feel safe during the COVID-19 pandemic.

The major limitation of this study is that it was carried out with the parents of orthodontic patients treated in our faculty. To ensure that these parents did not represent a biased sample, this study was conducted with the parents of patients whose orthodontic treatment was started prior to the COVID-19 pandemic and routine controls were continuing. Furthermore, conducting this study in a single center may have limited the application of the results to the whole population.

## Conclusion

COVID-19 has and will continue to have significant impacts on parents’ attitudes regarding pediatric dentistry practices. Although parents take COVID-19 seriously and know the transmission paths, many are hesitant to attend dental clinics for restorative dental treatment during the pandemic. Also, there are some differences in the attitudes and knowledge levels of parents towards dental treatments during the COVID-19 pandemic according to their educational level. Further studies are needed to evaluate the long-term impact of the COVID-19 pandemic on parents’ attitudes toward and knowledge about dental treatments.
